# A Case–Control Study on the Effects of Plasticizers Exposure on Male Fertility

**DOI:** 10.3390/ijerph20010235

**Published:** 2022-12-23

**Authors:** Lidia Caporossi, Paola Viganò, Enrico Paci, Silvia Capanna, Alessandra Alteri, Daniela Pigini, Giovanna Tranfo, Bruno Papaleo

**Affiliations:** 1 National Institute of Insurance against Accidents at Work-Department of Occupational and Environmental Medicine, Epidemiology and Hygiene, 00078 Monte Porzio Catone, Italy; 2 Unit of Obstetrics and Gynecology, San Raffaele Scientific Institute, 20132 Milan, Italy

**Keywords:** phthalates, bisphenol A, fertility, reprotoxicity

## Abstract

Male infertility is a serious concern for public health, and the possible role of exposure to plasticizers such as phthalates and bisphenol A in contributing to the condition is widely debated. We have herein enrolled 155 infertility cases attending an infertility center and 211 controls (fathers of a spontaneously conceived newborn) to investigate this issue. The urinary levels of seven phthalates and BPA were analyzed through HPLC/MS/MS. All data were statistically elaborated considering information about clinical situation, life habits, occupational activity, and, for cases, semen parameters (volume, sperm concentration, total count of spermatozoa, and sperm motility). Results showed significantly higher urinary concentrations for all the phthalates in cases compared to controls, except for monoethylphthalate and BPA. In total, 90.07% of cases had sperm motility lower than the WHO reference value (2010), while 53.69%, 46.31%, and 16.56% had sperm total number, concentration, and volume, respectively, out of the reference range. Regarding the possible source of exposure, the use of scents seems to be a significant source of DEP (diethylphthalate). When considering occupational settings, industrial workers, dental technicians, artisans, and farmers using chemicals showed higher risk (OR = 2.766, 95% CI 1.236–6.185), particularly in relation to DnBP (di-*n*-butyl phthalate) and DEHP (di-ethyl-hexyl phthalate) exposure. No clear quantitative correlation between specific plasticizers and sperm parameters could be demonstrated but these findings call for future studies about the risks associated with exposure to their mixture.

## 1. Introduction

Infertility is usually defined as the lack of ability to conceive a child after 12 months of unprotected intercourse [1]. It has been estimated that infertility affects about 37–70 million couples worldwide [2], and in 50 % of cases it can be ascribed to a male factor.

A meta-analysis of epidemiological data has shown that the quality of human semen has declined significantly over the last 40 years, with a severe reduction in sperm counts [3]. Similarly, there has been a decrease in sperm motility (15% in 17 years of follow-up) and an increase in morphological alterations in sperm (16% in 17 years of follow-up) [4]. 

During spermatogenesis, gametes are created daily in the seminiferous tubules, and spermatozoa, through the epididymis, subsequently reach the epididymal tail where they are collected until ejaculation [5]. All phases of the process are hormonally regulated, in particular by testosterone, 17-β-estradiol, follicle-stimulating hormone (FSH), and luteinizing hormone (LH). The mechanisms leading to male infertility are not completely clear, though changes in hormonal balance, particularly during sperm cell development, might affect the quality of spermatozoa. Risk factors that predispose an individual to male infertility can be various, ranging from congenital malformations to genetic problems, iatrogenic issues, and lifestyle factors [6]. Nevertheless, some environmental risk factors have been reported [7,8,9]. Specifically, possible exposure to endocrine disrupting chemicals (EDC) represents a theme of great concern among scientists [10], albeit not consistently [11]. Indeed, EDC, mostly phthalates and bisphenol A (BPA), may play a role due to possible negative effects on testicular steroidogenesis [12].

Phthalates are a group of chemicals that are used in numerous industrial processes [13]. The compounds with higher molecular weight, such as diethylhexyl-phthalate (DEHP) or di-*n*-octyl-phthalate (DnOP), are commonly used in different manufacturing industries (PVC, medical tools, food packaging, flooring products) in order to guarantee flexibility in plastic materials [14,15]. Compounds with lower molecular weight, such as diethylphthalate (DEP), di-*n*-butylphthalte (DnBP), and benzylbutylphthalate (BzBP), have commercial applications as solvents and fixatives in make-up and other products for beauty and wellness [16]. The commercial production of BPA worldwide is consistent; it is a constituent of polycarbonate plastics and epoxy resin products.

People can be exposed to phthalates and BPA mainly through dermal (i.e., body creams or personal care products) or ingestion (in cases of food contamination) routes [17,18,19], though the inhalation route is particularly important in occupational settings [20].

The health effects of phthalates have been widely documented in the literature [21]. Their ability to interact with the endocrine system seems to cause a variety of diseases, including thyroid and neurodevelopmental dysfunctions, asthma, endometriosis, type 2 diabetes, and breast cancer. Likewise, BPA has been known to be able to bind to steroid hormone receptors, in particular estrogen (α, β) receptors [22,23], and to exert disrupting effects on androgen synthesis [24]. Focusing on male reproductive health, phthalates have been suggested [25] to produce adverse effects on gonadal and non-gonadal tissues. These substances can impair spermatogenesis and alter the hormonal balance [26,27] between estradiol and testosterone, leading to possible testicular dysgenesis syndrome. Some authors [28] have found an association between increased protamine levels mediating sperm apoptosis and DEHP exposure. Others [29] have reported that phthalates can interfere with hormonal release at the hypothalamic level. Overall, these detrimental effects may result in reductions in sperm quality [30]. Phthalate exposure, especially DEHP, can also favor the production of reactive oxygen species [31] and the methylation of sperm DNA [32,33], causing acute damage to spermatozoa [31,32].

Considering the possible adverse effects of BPA, some evidence supports the anti-androgenic ability of this compound [34], with consequences on the motility of spermatozoa [35]. In vivo studies have highlighted the ability of BPA to induce testicular dysfunction [36] and testicular apoptosis [37,38,39]. More specifically, BPA exposure can induce a disorganization and degeneration of sperm cells [24], a fragmentation of pyknotic nuclei, and a significant reduction in sperm cell number [40,41].

These substances do not accumulate in the body; they are mainly excreted in the urine, with complete elimination within 24 h [42,43]. The compounds are metabolized to the respective monoester phthalates and a portion is oxidized [44,45,46]; BPA can be recovered in urine unaltered or metabolized into the respective glucuronide or disulfide form [47]. The urinary biomarkers have shown to be appropriate indicators for assessing human exposure to these chemicals.

On this basis, the aim of the present study was to investigate the possible differences in phthalate and BPA exposure between two male populations with different fertility status. Exposure was indeed compared between infertile patients and men with proven fertility, with a particular focus on the identification of potential occupational or lifestyle sources of exposure. The in-depth investigation of these sources, beside the possible differences in biological dosages, represents the innovative value of the present study.

## 2. Materials and Methods

### 2.1. Study Design and Recruitment of the Sample Population

This is a retrospective case–control study. The Institutional Review Board of the IRCCS San Raffaele, Scientific Institute in Milan, Italy, approved the research protocol (identification code 73/INT/2017).

Male partners from couples with infertility problems (defined as more than 12 months of unprotected intercourse without a pregnancy) (*n* = 274) were recruited at the Reproductive Center of San Raffaele Scientific Institute in Milan (Italy) between January 2018 and February 2019.

Inclusion criteria for enrollment as “cases” were infertility status and the presence of at least one abnormal semen parameter based on the recommendations of the World Health Organization (WHO, 2010). Participants were asked to collect a seminal fluid sample after at least 2 days of abstinence from intercourse.

Exclusion criteria included endocrine diseases with known etiology (e.g., adrenal disorders), testis injury, vasectomy, epididymitis, orchiditis, and previously diagnosed neoplasms that required specific chemotherapy and/or radiotherapy in the genital area. Subjects unable to provide a semen or urine sample and those with creatininuria out of the normal range set by the WHO for biological monitoring [48] were excluded.

Other variables were considered as possible confounding factors, including age, BMI, smoking, alcohol, and specific clinical situations potentially affecting the quality of the semen (e.g., varicocele or unresolved cryptorchidism).

After application of all the above-mentioned criteria, *n* = 155 subjects were included as cases.

Controls were recruited at the Obstetrics and Gynecology Unit of the IRCCS San Raffaele Hospital in Milan. The criteria observed for recruitment were the following: volunteers aged >18, male partners from couples with spontaneous conception (without hormonal therapy or assisted reproduction treatment), and a time to pregnancy lower than 12 months. From an initial number of 226 men who volunteered to participate in the study, after application of the inclusion criteria the sample was reduced to *n* = 211 subjects.

A questionnaire was administered by trained personnel to collect data from each participant on health status, life habits (diet, drugs, smoking, use of plastic containers for storage of fatty food, use of scents, or other possible source of phthalates), and working activity. Each participant also provided a urine spot sample so the concentration of phthalate metabolites and urinary BPA could be analyzed. Therefore, the study protocol was the same for cases and controls, with the only exception being semen collection and analysis, which was carried out only for cases. Finally, participants provided signed informed consent to participate in the study.

### 2.2. Urine Analysis

Analysis of the analytes of interest, phthalate metabolites and BPA in urine, was carried out using previously published methods [49,50] involving HPLC-MS/MS determination after pre-treatment of samples. Each urine sample was analyzed in duplicate; quantification was obtained using the linear regression equation identified from the calibration curve for the single metabolite, expressed in µg/L urine. The following metabolites were determined: mono-*n*-butyl phthalate (MnBP) with a limit of detection (LOD) of 0.8 µg/L, monoethyl phthalate (MEP) with an LOD of 3 µg/L, mono-benzyl phthalate (MBzP) with an LOD of 1.2 µg/L, mono-*n*-octyl phthalate (MnOP) with an LOD of 0.1 µg/L, mono(2-ethylhexyl)phthalate (MEHP) and mono(2-ethyl-5-hydroxyhexyl) phthalate (MEHHP), both metabolites of DEHP, with respective LODs of 0.15 and 0.05 µg/L, and urinary BPA with an LOD of 0.02 µg/L. In cases with values lower than the limit of detection (LOD), phthalate metabolite concentrations and urinary BPA concentrations were replaced with LOD/√2.

Urinary samples were also analyzed for their creatinine concentrations, which were determined using Jaffè’s method with alkaline picrate tests and spectrophotometric detection at a 490 nm wavelength [51]. Cases with values of urinary creatinine higher (more than 3.0 g/L) or lower (less than 0.3 g/L) than thresholds were excluded, as recommended by the American Conference of Governmental Industrial Hygienists (ACGIH) [52] and the WHO [48].

The final concentration of analytes was expressed in µg/g creatinine.

### 2.3. Semen Collection and Analysis

Each case subject provided a semen sample, which was collected at IRCCS San Raffaele Hospital in Milan (Italy) after a minimum of 2 days and a maximum of 7 days of ejaculatory abstinence.

After liquefaction in 30 min at 37 °C, the sample was characterized for volume, concentration, and motility of spermatozoa using a light microscope equipped with phase-contrast optics (Eclipse E200, Nikon) according to WHO criteria [53]. The total number of spermatozoa was then calculated considering the product of concentration and semen volume.

Sample volume was measured by weighing the sample in the container in which it had been collected, assuming the density of semen to be 1.0 g/mL.

The concentration of sperm was obtained using a Neubauer Improved (Bioanalytic GnbH, Umkirch, Germany) hemocytometer grid, with Gilson Microman M25, M50, or M250 positive displacement pipettes (Gilson UK, Luton, UK) used for dispensing the semen volume where appropriate for a dilution, and the analytical procedure was carried out according to WHO criteria [53].

The motility of spermatozoa was assessed at 37 °C and evaluated by counting at least 200 spermatozoa in a total of at least five fields in each replicate in order to achieve an acceptably low sampling error. Moreover, the average percentage of progressive, non-progressive, and immobile sperm was calculated. The SEMQUA (seminal QUAlity) studies checklist was used to improve the accuracy and clearness of the study [54].

An internal and external quality control program, in accordance with the guidelines of the European Society of Human Reproduction and Embryology (ESHRE) [54], was set up in the laboratory to evaluate possible interlaboratory differences.

The laboratory staff were trained in accordance with the ESHRE Special Interest Group in Andrology Basic Semen Analysis Course.

### 2.4. Statistical Analysis

Statistical analysis was conducted with SPSS^®^ software version 25 (IBM, Armonk, NY, USA). All concentration levels were elaborated using creatinine-corrected data.

Based on data distribution, non-parametric tests were used. Differences between the two groups were assessed using the Mann–Whitney test and the χ2 test for qualitative variables.

The Mann–Whitney test was also used to highlight case–control differences in metabolite values considered as a whole or stratified for possible exposure sources. Furthermore, a multivariate regression model was applied to evaluate the possible correlation between urinary metabolite concentrations and semen quality.

A further stratification of the values was carried out for working activities in order to point out possible differences between cases and controls using the Kruskal–Wallis test.

Finally, the Odds Ratios (ORs) were calculated based on raw data or adjusted for confounding factors (age, BMI, smoking, alcohol, and male morbidities) to express the risk of infertility associated with professional activities.

## 3. Results

### 3.1. Characteristics of the Study Population

The main characteristics of the enrolled population are shown in Table 1. The two groups of men were overall comparable; the only two parameters that represented a statistically significant difference were age (Mann–Whitney test *p* = 0.000, cases were on average slightly older than controls) and working activities (Mann–Whitney test *p* = 0.011). A potential difference in terms of chemical exposure at workplaces emerged between the two investigated populations.

### 3.2. Chemical Exposure and Elaboration

**Figure 1:**
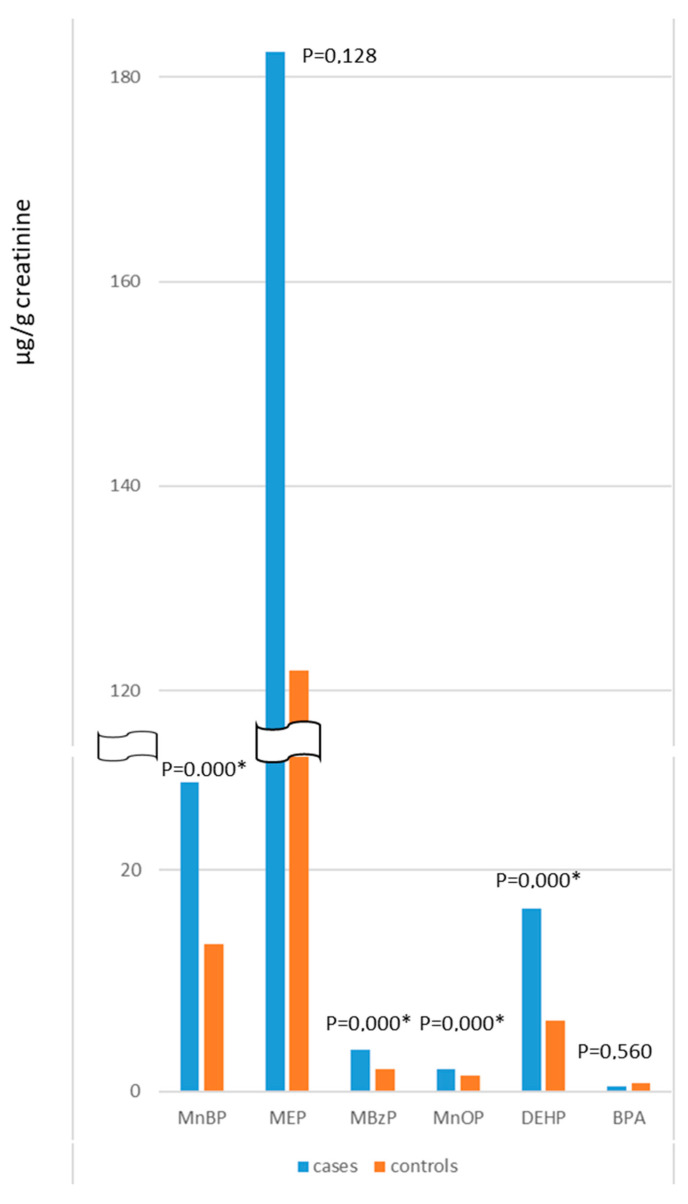
Comparison between the mean values of each metabolite in cases and controls. *p* values for Mann–Whitney tests are indicated. * significant value.

A visual comparison between the mean values of phthalate metabolites in cases and controls is presented in Figure 1. The analytical results of urinary analysis are reported in detail in Table 2, together with indications of significant differences between the two groups. Considering that data were not normally distributed, a non-parametric test was used (Mann–Whitney).

A statistically significant difference between cases and controls emerged for all the metabolites measured. Infertile men showed significantly higher urinary values for metabolite concentrations, with the only exceptions being MEP and BPA. Regarding MEP, even if the mean concentration value was higher for cases, the wide distribution of data and the high standard deviation did not reach statistical significance. Conversely, BPA urinary levels were similarly low in both groups.

Table 3 shows the seminal fluid parameters (volume, total number of spermatozoa, sperm concentration, and motility) in the population of infertility cases [53]. Sperm motility was the most significantly affected parameter, being below the WHO reference values in more than 90% of the infertile population. Total sperm count was abnormal in 53.69% of cases and sperm concentration in 46.3%. Conversely, semen volume appeared to be the less critical parameter, as only 16.56% of the studied population had values below the normal range. Table 4 shows the results of multivariate analysis considering the analyzed metabolites and seminal parameters. No specific correlations were found for any of the studied metabolites in relation to any of the semen parameters after adjustment for confounding factors (age, smoke, BMI, alcohol use, genital pathologies) and considering a significance level of 95%.

In consideration of previously published results [50] describing possible exposure risks, the same data are shown in Table 5 after a stratification based on the habits of using scents and of using plastic containers for fat food storage, suspected to be possible relevant sources. A significantly more frequent use of perfumes was associated with an increased exposure to DEP, and therefore with a higher urinary MEP level. This evidence did not emerge for any of the other plasticizers. No significant results were conversely found in relation to the habit of using plastic containers for the storage of fat foods.

In order to clarify whether certain types of working activities could be associated with exposure to a specific plasticizer, a stratification of data in relation to this aspect was carried out (Table 6). This distribution of the data did not actually show any specific exposure for the occupational activities reported by the population under study (Kruskal–Wallis test).

Finally, to better investigate the possible role of occupational activity on reproductive health, ORs for infertility were calculated considering differences between cases and controls (Table 7). This risk index represents the “cumulative” effects of occupational risk factors. From calculation of these indices, we found that activities that involve remaining for long periods of time in sitting positions were definitely at lower risk in terms of reproductive health issues, whereas professional activities involving possible exposure to chemical agents to some degree presented a higher risk as expected.

## 4. Discussion

The results obtained herein demonstrate significantly higher urinary concentrations for all the phthalates analyzed in cases compared to controls, except for MEP and BPA. In particular, the infertile population demonstrated, on average, values for MnBP, MBzP, and DEHP that were almost double those of controls, as well as a 50% higher value for MnOP.

The analytes were detectable in most of the samples, except for MBzP, which was measured in about half of the samples. It is noteworthy that DEHP metabolites could be detected in all samples, despite DEHP being one of the phthalates with increased marketing restrictions due to its well-known toxicity.

Comparing our concentrations of urinary metabolites with data in the literature, sometimes significant differences emerge that are probably related to geographical variability. Specifically, the mean value for MEP detected in the present study (182.47 µg/g creatinine) is comparable to findings collected in other Western countries, such as the US (200.96 µg/L) [55] and Sweden (190 µg/L) [56], while it is considerably higher than that reported in some Asian populations (15.2 µg/L and 18.8µg/L) [57]. The urinary concentrations of this metabolite, known to be strongly linked to the use of personal care products, always present important variability. According to the present findings, the frequent use of scents represents the only route of exposure to MEP clearly identified in daily life (*p* = 0.000). DEP is used in numerous types of formulations during perfume production. A previous study [58] analyzed 47 branded perfumes and highlighted the presence of DEP in all samples, with concentration levels ranging from 1621.6 ppm to 23,649.2 ppm. In general, the high concentration of MEP detected in urine samples is due to the absence of specific laws that place restrictions on the use of the compound in industrial processes and marketing, although the health effects associated with human exposure are not yet clearly understood.

For the other metabolites, the order of magnitude seems to be similar to those previously reported, albeit with some variability in values. Published values for urinary MBzP concentrations range from 0.2 to 22.0 µg/L, while MnBP levels range from 15.28 to 147 µg/L [55,56,57,59]. Our findings are quite similar in both cases, with 3.73 µg/g creatinine and 27.96 µg/g creatinine for MBzP and MnBP, respectively.

For the metabolites of DEHP, published values range from 17 to 40 µg/L, comparable with our results (16.56 µg/g creatinine). For MnOP, it is difficult to find historical data because it has been rarely assessed. Pan et al. [57] failed to detect the metabolite, finding concentrations lower than the LOD.

To the best of our knowledge, we were the first to have clearly demonstrated higher levels of several metabolites in cases compared to controls, which is in disagreement with some previous studies. In a previous report [60], the assessment of 16 different free and conjugated metabolites led to the detection of significant differences in only MEP (43.4 vs. 28.9 µg/g creatinine) and MEHP (3.3 vs. 2.5 µg/g creatinine), with values lower in order of magnitude than those found in our study for MEP. Den Hond et al. [61] did not observe statistical differences between cases and controls when analyzing MEHP (2.9 vs. 2.6 µg/L), MEP (40.5 vs. 49.9 µg/L), MnBP (20.7 vs. 18.9 µg/L), MBzP (4.6 vs. 4.5 µg/L), and BPA (1.7 vs. 1.5 µg/L). Discrepancies when compared to our study could be explained by the lower number of subjects previously considered (80 cases and 40 controls), as well as the different criteria used for the selection of controls (who were recruited from a fertility center among men with a higher total motility count).

Notably, most of the investigations did not normalize urinary concentrations for creatinine levels. This choice implies, in general, the imprecision of the data obtained, linked to a possible bias due to the dilution of the analytical matrix. For this reason, comparison to our data can be correctly performed only in terms of orders of magnitude and not exact levels.

In the population of infertile men under study, the sperm motility parameter was found to be lower than the reference value for 90.07% of subjects, thus confirming it to be the most critical parameter. This was followed by the total number of spermatozoa (in 53.69% of cases it was lower than the reference value), sperm concentration (in 46.31% of cases it was out of range), and volume (in only 16.56% of cases was it abnormal).

Previous research has documented a correlation between exposure to specific phthalates and alterations in semen quality. In particular, MnBP has been positively associated with a significant decrease in semen concentration [62,63] and sperm count (*p* < 0.05). Along this line, an increase in MEHP levels has been associated with a significant increase in “abnormal heads” of spermatozoa (*p* < 0.01) [62], as well as with an increase in oxidative stress at the sperm level [31].

Other authors [63] have identified a significant relationship between MBzP levels and MEHHP levels (but not of other metabolites of DEHP) and reduced sperm counts, while exposure to DEHP [56] has been associated with lower sperm motility [57,64]. Another study [55] confirmed the involvement of MBzP in reduced sperm concentration and motility (β = −1.47, 95% CI: −2.61, −0.33). Finally, a recent cross-sectional study [50] from our group suggested a correlation between exposure to MEP and reductions in sperm concentration (β= 0.303, 95% CI: 0.246/0.035).

Overall, there is good evidence to suggest that exposure to phthalates may be involved in sperm alterations and may therefore affect reproductive health, although it has to be admitted that a clear quantitative relationship between exposure and the reduced quality of seminal parameters is not easily assessable [65].

Indeed, when we adjusted the variables for confounding factors, the correlation be-tween urinary analyte levels and semen parameters lost its statistical significance, thus limiting the possibility of clarifying whether one specific substance rather than another was more involved.

The combination of these data suggests that the risk should be addressed in terms of multiple exposure, since exposure to several phthalates at the same time could lead to a cumulative or even synergistic effect and result in clearer detectable changes. In this context, we are encouraged by the fact that all cases had higher values for the measured analytes compared to controls, which is not a secondary aspect.

By studying workplaces and possible sources of exposure, it emerged that groups engaged in activities involving exposure to chemical agents were, as expected, at higher risk. Farmers, artisans, dental technicians, and industrial workers showed an OR = 2.766 (95% CI 1.236–6.185) for infertility, and exposure to DnBP and DEHP in particular seems to be highly involved. Other working activities did not have an impact on male reproductive health.

A limit of this study is related to the determination of analytes on a single spot of urine, which allowed for exposure during the previous 24–48 h to be assessed. Another limitation is represented by the evaluation of exposure related to living and working environments relying exclusively on self-reported data. We could not directly verify the absence or presence of specific sources of exposure.

On the other hand, a very strict selection of cases and controls represents a strength of the investigation, which allowed important differences to be detected and helped confirm data from the literature. The frequent use of perfumes has been identified as a life habit that possibly represents a route of significant exposure to DEP. Furthermore, the focus on working environments was another point in favor of the validity of the present study.

## 5. Conclusions

Male infertility is a serious concern for public health because of its widespread prevalence and serious consequences. Numerous factors could lead to reproductive problems, both in environmental and occupational settings. Experimental data both in vitro and in vivo highlighted the involvement of phthalates in negatively affecting spermatogenesis and increasing oxidative stress and DNA damage, resulting in poor quality semen [66,67].

The results of the present study confirmed higher exposure to several phthalates in a population of infertile men compared to a fertile population. Even if a correlation between specific metabolites and semen parameters did not emerge, we cannot exclude the impact of multiple exposure on the observed health effects, particularly on sperm motility (which is the most critical parameter).

Regarding the route of environmental exposure, the use of scents was shown as a source of DEP exposure, confirming previous observations [50].

Considering possible occupational exposure, for men working in industrial plants, artisans, or other professions implying the use of chemicals, such as dentists, technicians, and farmers, evidence of the type of workplace contributing to infertility status emerged (OR = 2.766, 95% CI 1.236–6.185).

These findings call for novel investigations regarding the context of occupational settings in order to elucidate the specific environmental exposure involved, with the final aim of protecting workers from reproductive risks.

## Figures and Tables

**Table 1:** Main characteristics of the population under study.

Characteristics	Cases (155)	Controls (211)	*p* Value
**Age (range)**	40.4 (30–67)	36.1 (22–58)	0.000 *
**BMI ^1^ (% of subjects in the class)**			
Underweight	0.0	0.5	0.490
Normal	52.3	50.7	
Overweight	36.8	39.8	
Obese	9.6	6.2	
Missing	1.3	2.8	
**Actually smokers (%)**	27.7	22.7	0.368
**Previously smokers (%)**	23.9	26.5	
**Alcohol consumption (%)**			
Daily	14.8	19.0	0.722
Weekly	56.1	49.3	
Monthly	18.1	18.5	
Never	9.0	10.9	
Missing	1.9	2.4	
**Area of residence (%)**			
Urban	76.1	87.2	0.084
Rural	11.0	8.5	
Coast	3.9	0.9	
Industrial	1.9	0.5	
Other	3.2	1.4	
**Use of plastic containers for fat food storage (%)**			
Never	20.0	14.2	0.484
Daily	22.6	24.2	
Weekly	36.8	43.1	
Monthly	17.4	16.6	
Missing	3.2	1.9	
**Eating canned food at least weekly (%)**	46.5	45.5	0.677
**Eating soya products at least weekly (%)**	14.8	13.8	0.922
**Use of scents at least weekly (%)**	62.6	74.0	0.095
**Use of hair spray at least weekly (%)**	5.1	10.4	0.186
**Working activity (%)**			
Administrative workers or those working at a computer for more than 20 h/week, drivers	41.9	56.4	0.011 *
Activity with possible chemical exposure (farmers, artisans, dental technicians)	16.1	8.5	
Nurses, physicians, health operators	3.2	4.3	
Soldiers, policemen	6.5	2.8	
Other	26.5	19.0	
Missing	5.8	9.0	

^1^ BMI: body mass index; * significant difference; Mann–Whitney test for continue variables; χ^2^ test for qualitative variables.

**Table 2:** Analytical results (µg/g creatinine) of urinary metabolites of phthalates and BPA.

Parameters	MnBP ^1^		MEP ^2^		MBzP ^3^		MnOP ^4^		DEHP ^5^		BPA ^6^	
	Cases	Controls	Cases	Controls	Cases	Controls	Cases	Controls	Cases	Controls	Cases	Controls
*n* subjects	155	211	155	211	155	211	155	211	155	211	108	140
Arithmetic mean	27.96	13.33	182.47	121.93	3.73	2.03	2.01	1.41	16.56	6.40	0.47	0.69
Median	15.01	8.71	26.85	30.69	2.48	0.84	1.05	0.71	7.90	3.77	0.12	0.12
Standard deviation	43.86	16.88	676.54	335.21	5.37	3.63	3.23	3.17	27.28	9.41	1.07	1.28
5° percentile	2.02	0.71	1.02	1.50	0.46	0.18	0.05	0.16	1.24	0.56	0.03	0.00
95° percentile	105.17	39.95	721.87	516.95	11.55	6.19	7.84	4.55	69.02	22.21	2.69	4.08
*p* value (Mann–Whitney test)	0.000 *		0.128		0.000 *		0.007 *		0.000 *		0.560	
LOD ^7^ (µg/L)	0.8		3.0		1.2		0.1		0.15 MEHP ^8^ 0.05 MEHHP ^9^		0.02	
% > LOD	98.7	94.3	100.0	87.2	66.4	44.5	91.6	99.5	100.0	100.0	96.0	95.0

^1^ MnBP—mono-*n*-butyl phthalate; ^2^ MEP—mono ethyl phthalate; ^3^ MBzP—monobenzyl phthalate; ^4^ MnOP—mono-*n*-octyl phthalate; ^5^ DEHP—di-(2-ethylhexyl) phthalate, molar sum of two metabolites; ^6^ BPA—bisphenol A; ^7^ LOD—limit of detection; ^8^ MEHP—mono(2-ethylhexyl) phthalate; ^9^ MEHHP—mono(2-ethyl-5-hydroxyhexyl) phthalate. * Significant value.

**Table 3:** Results of seminal parameters in the cases population.

Parameters	Volume (mL)	Total Number of Spermatozoa (*n*)	Concentration (*n*/mL)	Motility (%)
*n* subjects	151	149	149	151
Arithmetic mean	2.36	54.6 × 10^6^	24.5 × 10^6^	21.42
Median	2.50	36.0 × 10^6^	16.0 × 10^6^	20.00
Standard deviation	1.08	63.6 × 10^6^	25.9 × 10^6^	13.86
5° percentile	0.30	0.1 × 10^6^	0.8 × 10^6^	1.00
95° percentile	4.00	197.5 × 10^6^	81.0 × 10^6^	50.00
Reference values (WHO)	1.5	39.0 × 10^6^	15 × 10^6^	40.00
% results< WHO reference values	16.56	53.69	46.31	90.07

**Table 4:** Multivariate analysis among urinary metabolites and seminal fluid parameters, after adjustment for confounding factors (age, smoke, BMI, alcohol use, genital pathologies).

Variables	Semen Volume			Semen Concentration			Spermatozoa Motility			Total Number of Spermatozoa		
	β ^1^	95% CI	*p* Value	β	95% CI	*p* Value	β	95% CI	*p* Value	β	95% CI	*p* Value
MnBP	0.140	−0.001/0.008	0.112	−0.066	−0.144/0.065	0.453	−0.082	−0.080/0.030	0.349	−0.088	−0.392/0.131	0.324
MEP	0.114	0.000/0.000	0.189	−0.126	−0.012/0.002	0.143	−0.092	−0.006/0.002	0.281	−0.127	−0.030/0.004	0.146
MBzP	0.050	−0.025/0.045	0.574	−0.057	−0.148/0.580	0.517	−0.065	−0.619/0.282	0.461	−0.090	−3.264/1.062	0.316
MnOP	0.146	−0.010/0.104	0.104	−0.011	−1.985/0.826	0.416	−0.031	−0.867/0.606	0.727	−0.062	−4.741/2.322	0.499
DEHP	0.119	−0.002/0.011	0.194	−0.050	−0.214/0.122	0.588	−0.122	−0.147/0.028	0.181	−0.020	−0.468/0.376	0.830
BPA	0.184	−0.042/0.411	0.109	0.160	−9.010/1.707	0.179	0.118	−1.417/4.240	0.324	−0.008	−11.591/10.881	0.950

^1^ β: standardized regression coefficient.

**Table 5:** Urinary levels of phthalate metabolites and BPA (average ± standard deviation in µg/g creatinine), stratification for the use of scents and the use of plastic containers to store fat food, and comparison through Kruskal–Wallis tests.

Frequency of Use	MnBP ^1^	MEP ^2^	MBzP ^3^	MnOP ^4^	DEHP ^5^	BPA ^6^
**Use of scents**						
Daily	22.64 ± 41.20	181.89 ± 622.64	2.93 ± 5.01	1.53 ± 2.40	10.11 ± 15.77	0.58 ± 1.17
Weekly	19.00 ± 20.52	139.14 ± 255.78	2.57 ± 3.46	2.12 ± 3.45	15.61 ± 32.46	0.76 ± 1.36
Monthly	12.23 ± 10.70	139.85 ± 534.58	2.60 ± 4.41	1.66 ± 2.63	7.79 ± 8.13	0.86 ± 1.80
Never	15.92 ± 19.29	87.49 ± 379.29	2.69 ± 4.35	1.55 ± 4.48	8.55 ± 15.13	0.43 ± 0.73
*p* value Kruskal–Wallis test	0.437	0.000 *	0.952	0.102	0.591	0.181
**Use of plastic containers for fat food**						
Daily	21.93 ± 31.83	104.70 ± 207.09	3.25 ± 5.59	2.36 ± 5.42	12.99 ± 24.06	0.51 ± 1.03
Weekly	19.22 ± 28.59	193.68 ± 712.74	2.50 ± 2.89	1.45 ± 1.75	9.08 ± 12.89	0.62 ± 1.22
Monthly	15.87 ± 17.38	182.41 ± 479.85	2.92 ± 4.21	1.70 ± 3.04	13.69 ± 30.11	0.55 ± 1.05
Never	21.52 ± 49.65	71.17 ± 126.23	2.84 ± 6.38	1.21 ± 1.59	8.49 ± 13.39	0.76 ± 1.54
*p* value Kruskal–Wallis test	0.965	0.736	0.884	0.802	0.774	0.580

^1^ MnBP—mono-*n*-butyl phthalate; ^2^ MEP—mono ethyl phthalate; ^3^ MBzP—monobenzyl phthalate; ^4^ MnOP—mono-*n*-octyl phthalate; ^5^ DEHP—di-(2-ethylhexyl) phthalate, molar sum of two metabolites; ^6^ BPA—bisphenol A. * Significant value.

**Table 6:** Urinary levels of phthalate metabolites and BPA (average ± standard deviation in µg/g creatinine), stratification for working activity, and comparison through Kruskal–Wallis tests.

Working Activity	MnBP ^1^	MEP ^2^	MBzP ^3^	MnOP ^4^	DEHP ^5^	BPA ^6^
Administrative workers, drivers	17.89 ± 25.52	174.62 ± 640.18	2.63 ± 3.47	1.27 ± 1.52	8.67 ± 13.61	0.56 ± 1.21
Farmers, artisans, dental technicians	16.45 ± 18.17	67.11 ± 139.11	2.66 ± 5.36	3.90 ± 7.53	21.47 ± 41.87	0.77 ± 1.33
Nurses, physicians, health operators	20.35 ± 26.56	28.45 ± 44.96	3.19 ± 4.98	1.36 ± 2.11	14.18 ± 28.38	0.51 ± 0.49
Soldiers, policemen	34.56 ± 63.73	79.48 ± 85.57	2.53 ± 2.70	1.98 ± 2.64	11.66 ± 14.39	0.21 ± 0.41
Other	24.04 ± 44.90	154.39 ± 433.20	3.33 ± 6.45	1.50 ± 2.45	9.55 ± 12.71	0.66 ± 1.21
*p* value Kruskal–Wallis test	0.608	0.106	0.838	0.174	0.272	0.053

^1^ MnBP—mono-*n*-butyl phthalate; ^2^ MEP—mono ethyl phthalate; ^3^ MBzP—monobenzyl phthalate; ^4^ MnOP—mono-*n*-octyl phthalate; ^5^ DEHP—di-(2-ethylhexyl) phthalate, molar sum of two metabolites; ^6^ BPA—bisphenol A.

**Table 7:** Odds Ratios for working activity and for possible sources of exposure and risk, adjusted for age, BMI, smoke, alcohol, and specific pathologies.

Working Activity	Cases	Controls	OR	95% CI	*p* Value
Administrative workers, every activity that requires working on a computer for more than 20 h/week, drivers	65	119	0.534	0.321–0.888	0.016 *
Activity with possible chemical exposure (farmers, artisans, dental technicians)	25	18	2.766	1.236–6.185	0.013 *
Nurses, physicians, health operators	5	9	0.306	0.084–1.118	0.073
Soldiers, policemen	10	6	3.233	0.976–10.711	0.055
Other	49	58	1.220	0.699–2.129	0.485

* Significant value.

## Data Availability

The data presented in this study are available on request from the corresponding author. The data are not publicly available due to privacy and ethical reasons.
